# Multiple roles for a novel RND‐type efflux system in *Acinetobacter baumannii* AB5075

**DOI:** 10.1002/mbo3.418

**Published:** 2016-10-19

**Authors:** Kyle A. Tipton, Marjan Farokhyfar, Philip N. Rather

**Affiliations:** ^1^ Department of Microbiology and ImmunologyEmory University School of Medicine AtlantaAtlantaGAUSA; ^2^ Research ServiceAtlanta VA Medical CenterDecaturGAUSA; ^3^ Emory Antibiotic Resistance CenterEmory University School of MedicineAtlantaGAUSA

**Keywords:** *Acinetobacter*, phase variation, RND efflux system

## Abstract

Colony opacity phase variation in *Acinetobacter baumannii* strain AB5075 is regulated by a reversible high‐frequency switch. Transposon mutagenesis was used to generate mutations that decreased the opaque to translucent switch and a gene encoding a predicted periplasmic membrane fusion component of a resistance–nodulation–cell division (RND)‐type efflux system was isolated. This gene was designated *arpA* and immediately downstream was a gene designated *arpB* that encodes a predicted membrane transporter of RND‐type systems. A nonpolar, in‐frame deletion in *arpA* resulted in a 70‐fold decrease in the opaque to translucent switch. An *arpB::Tc* mutant exhibited a 769‐fold decrease in the opaque to translucent switch. However, the translucent to opaque switch was largely unchanged in both the *arpA* and *arpB* mutants. The *arpA* and *arpB* mutants also exhibited increased surface motility in the opaque form and the *arpB* mutant exhibited increased susceptibility to aminoglycosides. The *arpA* and *arpB* mutants were both attenuated in a *Galleria mellonella* model of virulence. A divergently transcribed TetR‐type regulator ArpR was capable of repressing the *arpAB* operon when this TetR regulator was overexpressed. The *arpR* gene was also involved in regulating the opaque to translucent switch as an in‐frame *arpR* mutation decreased this switch by 1,916‐fold.

## 1 Introduction

*Acinetobacter baumannii* is a Gram‐negative pathogen typically associated with infections in hospital settings (Bergogne‐Berezin & Towner, 1996; Gootz & Marra, 2008; Joly‐Guillou, 2005; Peleg, Seifert, & Paterson, 2008). Although most *A. baumannii* infections are seen in immunocompromised patients or those with severe injuries, community acquired infections and infections in otherwise healthy patients have increased in recent years (Antunes, Visca, & Towner, 2014; Charnot‐Katsikas et al., 2009; Guerrero et al., 2010; Lowman, Kalk, Menezes, John, & Grobusch, 2008). This development, combined with the increasing frequency of multidrug resistance, has made *A. baumannii* an extremely problematic pathogen for clinicians to treat and mortality rates for these infections has approached 70% (Lee, Chen, Wu, Huang, & Chiu, 2014). It has become widely recognized that new therapies are needed to help combat these infections (Gootz & Marra, 2008; Hujer et al., 2006; Joly‐Guillou, 2005; Scott et al., 2007).

Members of the resistance–nodulation–cell division (RND) class of efflux systems in Gram‐negative bacteria are composed of three proteins: an inner membrane transporter, an outer membrane protein that serves as a pore, and a periplasmic adapter protein that interacts with both the inner and outer membrane proteins to form a conduit for the extrusion of small molecules. RND‐type systems typically capture toxic compounds or metabolites and remove them from the cell, and because of this function they can be involved in resistance to antibiotics, disinfectants, and heavy metals (Alvarez‐Ortega, Olivares, & Martinez, 2013; Anes, McCusker, Fanning, & Martins, 2015; Delmar, Su, & Yu, 2014; Magnet, Courvalin, & Lambert, 2001; Routh et al., 2011; Venter, Mowla, Ohene‐Agyei, & Ma, 2015). They have also been shown to have roles in virulence, resistance to host antimicrobial peptides, and in cellular homeostasis by removing excess metabolites (Helling et al., 2002; Warner, Folster, Shafer, & Jerse, 2007). *Acinetobacter baumannii* possesses a number of RND‐type efflux systems that have roles in antibiotic resistance, virulence, and biofilm formation (Damier‐Piolle, Magnet, Bremont, Lambert, & Courvalin, 2008; Magnet et al., 2001; Yoon et al., 2015), and these systems are reviewed in Coyne, Courvalin, and Perichon (2011). The expression of these genes in *A. baumannii* is often regulated at the level of transcription by activator and/or repressor proteins (Lin, Lin, & Lan, 2015; Marchand, Damier‐Piolle, Courvalin, & Lambert, 2004; Rosenfeld, Bouchier, Courvalin, & Perichon, 2012).

Recently, our laboratory described a high‐frequency switch that results in the interconversion between opaque and translucent colony opacity phenotypes (Tipton, Dimitrova, & Rather, 2015). This switch is mediated by an unknown mechanism, but is stimulated at high cell density. Unique phenotypes are associated with each colony variant. For example, the opaque variants are more motile, highly virulent, and exhibit higher levels of resistance to aminoglycosides (Tipton et al., 2015). In contrast, translucent variants are more adept at forming biofilms on both polystyrene and glass.

To begin understanding the mechanism underlying this high‐frequency colony opacity switch, transposon mutagenesis was used to generate mutations in strain AB5075 that greatly reduced the frequency of phase variation from opaque to translucent. One mutant revealed a role for a previously uncharacterized RND‐type system in this process. Mutations in the genes encoding this RND system significantly decreased phase variation in the opaque to translucent direction, but had little to no effect on phase variation in the translucent to opaque direction. Moreover, mutations inactivating this RND system were pleiotropic and resulted in altered surface motility, aminoglycoside resistance, and virulence in a *Galleria mellonella* waxworm model.

## 2 Experimental Procedures

### 2.1 Bacterial strains, plasmids, and growth conditions

Both *A. baumannii* and *Escherichia coli* were grown in modified Luria Broth containing 10 g tryptone, 5 g yeast extract, and 5 g NaCl per liter. For screening opaque and translucent colonies of AB5075, LB was prepared at 0.5× of the normal concentration with 8 g agar per liter. *Escherichia coli* transformants were selected with chloramphenicol (25 μg/ml), ampicillin (200 μg/ml), or kanamycin (20 μg/ml) when appropriate. *Acinetobacter baumannii* AB5075 transformants were selected with tetracycline (3 μg/ml). Plamsid pEX18Tc was used for allelic replacement in AB5075 (Hoang, Karkhoff‐Schweizer, Kutchma, & Schweizer, 1998). For all experiments involving opaque and translucent variants, cells were obtained from freezer stocks that were grown to low density and contained <0.5% of the opposite cell type. Strain AB00075 *arpB::Tc* was obtained from the *A. baumannii* transposon insertion library maintained by Dr. Colin Manoil's laboratory at the University of Washington (Gallagher et al., 2015).

### 2.2 Phase variation assays

Freezer stocks of each strain that were verified as 99.99% of a single colony type were grown in LB broth to an OD of 1.8–1.9. Colonies were resuspended in LB broth and serial dilutions were plated on 0.5× LB, 0.8% agar plates. After overnight growth, the total number of viable cells per ml was determined and the number of opaque or translucent colonies present was determined on plates with at least 200 colonies/plate by using oblique light to illuminate the colonies.

### 2.3 Transposon mutagenesis

A culture of the *A. baumannii* AB5075 opaque variant was grown in 25 ml of LB broth at 37°C with vigorous shaking. Cells were harvested from cultures at an OD_600_ ~0.5 by centrifugation at 4°C for 10 min. Pellets were washed twice with 10% glycerol to prepare electrocompetent cells. Transposon (0.1 pmole) and transposase (1 unit) components of an EZ‐Tn5 <TET‐1> kit (Epicentre Biotechnologies) were combined with 10% glycerol and incubated at 37°C for approximately 45 min. Transposome complex mixture was then cooled on ice prior to electroporation. Aliquots of 1.25 μl transposome mixture and 1 μl of TypeOne Restriction Inhibitor (Epicentre Biotechnologies) were electroporated into 60 μl of competent AB5075 O cells at 2.5 kV. Following electroporation, 1 ml of room temperature LB broth was added following electroporation. This cell suspension was transferred to tubes and incubated at 37°C with shaking for 1 hr. Mutagenized cells were plated in 100 μl aliquots on 0.5× LB, 0.8% agar plates supplemented with 3 μg/ml tetracycline to select for insertional mutants and plates were incubated at 37°C. Colonies were evaluated at 24 and 48 hr postplating for altered colony morphology or reduced colony sectoring when viewed under a dissecting scope with oblique illumination from below. Putative mutants were restreaked on 0.5× LB, 0.8% agar with Tet^3^ to ensure colony morphology was stable and that picked colonies were pure. Identification of insertion sites was accomplished by rescue cloning of the tetracycline resistance gene and DNA sequencing of plasmids generated from rescue cloning.

### 2.4 Construction of in‐frame deletions in *arpA* and *arpR*

An in‐frame deletion in *arpA* was generated as described previously (Hoang et al., 1998) from AB5075 genomic DNA by PCR amplification of two approximately 1 kb fragments containing small portions of the *arpA* coding region using the primers arpA Up‐1 (5′‐AAAAAGGATCCATACTACGTTGTACCGCTAC‐3′), arpA Up‐2 (5′‐GTGAAAATTCAGGGAGCCA‐3′), arpA Down‐1 (5′‐TTGTTCGTGAAGTGTGGTT‐3′), and arpA Down‐2 (5′‐AAAAAGGATCCCACCTAATAAATTGCCAAGTAAGC‐3′). Oligonucleotide primers arpA Up‐1 and arpA Down‐2 were engineered to contain *BamH*I restriction sites at the 5′ end. The up‐ and downstream fragments were ligated together to produce an approximately 2‐kbp fragment containing the Δ*arpA* allele which was subsequently gel purified. The Δ*arpA* allele contains an in‐frame deletion in the *arpA* coding sequence corresponding to amino acids 48–359 of the 366 (85% of coding region). Purified fragment was reamplified with outer primers (arpA Up‐1 and arpA Down‐2) and gel purified. The Δ*arpA* fragment and pEX18Tc were digested with *BamH*I and gel purified. Digested fragments were then ligated and transformed into competent *E. coli* DH5α cells. To transfer the mutant alleles to the chromosome of *A. baumannii* AB5075, the suicide vector containing the in‐frame *arpA* deletion was electroporated into competent AB5075 cells which had been grown overnight in LB and washed with 300 mM sucrose as described previously (Choi & Schweizer, 2006). Integrants were selected on LB + tetracycline at 5 μg/ml. Counterselection to select for excision of the integrated plasmid was carried out at room temperature on LB without NaCl supplemented with 10% sucrose. Potential mutants were screened by PCR and confirmed by DNA sequencing.

The *arpR* in‐frame deletion allele was generated from AB5075 genomic DNA by PCR amplification (Phusion Hot‐Start Polymerase, Thermo Scientific) of two approximately 1 kbp fragments containing small portions of the *arpR* coding region. Oligonucleotide primers arpR Up‐1 (5′‐AAAAAGGATCCCGTGATAACCACAATACTTC‐3′) and arpR Down‐2 (5′‐AAAAAGGATCCATGACATTAGTTTGAGTCGA‐3′) were engineered to contain *BamH*I restriction sites at the 5′ end and were paired with arpR Up‐2 (5′‐CTCAAATATCGGCATTAAACC‐3′) and arpR Down‐1 (5′‐ATTAACTGTTTGCACGAAAC‐3′), respectively. The up‐ and downstream fragments were ligated (Fast‐Link DNA Ligation Kit, Epicentre Biotechnologies) together to produce an approximately 2 kbp fragment containing the *ΔarpR* allele which was subsequently gel purified (UltraClean 15 DNA Purification Kit, MoBio Laboratories). The *ΔarpR* mutant allele contains an in‐frame deletion corresponding to amino acids 6–202 of the 207 aa protein (94% of protein sequence). Purified deletion fragment was reamplified with outer primers (acrR Up‐1 and Down‐2) and gel purified. The *ΔarpR* fragment and pEX18Tc were digested with *BamH*I and gel purified. Digested fragments were ligated and transferred into competent *E. coli* EC100D Transformax cells (Epicentre Biotechnologies). This ligation produced the suicide vector pΔarpR/EX18Tc. This construct was then used to create an in‐frame deletion in *arpR* as described above for *arpA*.

### 2.5 Complementation of the *arpA* mutant

The full‐length *arpA* allele including 91 bp of sequence upstream from the ATG start codon and 42 bp downstream from the stop codon were amplified from *A. baumannii* AB5075 genomic DNA by PCR with the following oligonucleotide primers: 5′‐CCTTATTGTTCAGTGCCCAT‐3′ and 5′‐GTGCCGTCGGGTATATTAATTA‐3′. Primers were phosphorylated prior to PCR amplification to add 5′ phosphates with T4 polynucleotide kinase (New England Biolabs). The *arpA* fragment was gel purified and ligated with the shuttle vector pWH1266 (Hunger, Schmucker, Kishan, & Hillen, 1990) which had been digested with ScaI and treated with recombinant shrimp alkaline phosphatase (New England Biolabs). The ligation mixture was introduced into *E. coli* EC100D Transformax cells (Epicentre) via electroporation and clones were screened for tetracycline resistance and ampicillin sensitivity. The following plasmid pArpA was confirmed by DNA sequencing prior to introduction into *A. baumannii*. Competent *A. baumannii* AB5075 opaque and Δ*arpA* opaque cells were prepared by washing cells from overnight LB plates in dH_2_O two times. pArpA plasmid was electroporated into competent cells and transformants were selected on LB + tetracycline at 5 μg/ml. For switching frequency assays, overnight starter cultures of LB + tetracycline (3 μg/ml) were inoculated with AB5075 or Δ*arpA* harboring pWH1266 or pArpA and incubated at room temperature overnight without shaking. Strains were subcultured into LB without antibiotics and incubated at 37°C with vigorous shaking. CFU/ml and number of translucent colonies (switching frequency) were quantified at OD_600_ 0.7 and 1.7.

### 2.6 Preparation of conditioned media

To prepare conditioned media, cells were grown in 25 ml LB cultures at an optical density of 1.7. At this time, aliquots were restreaked to verify that the cultures remained at least 95% opaque or translucent. Cells were removed by centrifugation and the resulting media was adjusted to pH 7.0 with HCl and filter sterilized by passing through a 0.22‐μm filter. Aliquots were frozen at −80°C and used within 2 weeks. To grow cells in conditioned media, a 2‐ml aliquot was thawed and a 10× concentrate of tryptone and yeast extract (TY) was added back to a final concentration of 0.25×. Cells were grown by shaking at 270 rpm at 37°C to an optical density of 1.1 and dilutions were then plated to determine phase variation frequencies. Growth of cells in the LB control was done at 0.25× LB, a concentration that gave a similar growth rate as the TY‐supplemented conditioned media.

### 2.7 *Galleria mellonella* infections

*Galleria mellonella* larvae between 200 and 250 mg were utilized for infection studies. *Acinetobacter baumannii* strains were grown in LB broth at 37°C with shaking to an OD_600_ ~0.5. Cultures were serially diluted in LB broth and plated to determine CFU/ml for each bacterial strain. Strains diluted to 10^−2^ were chilled on ice prior to injection into larvae. Four μl aliquots of each strain were injected into the hemolymph of *G. mellonella* larvae (10 larvae per strain in three replicates, ~30 total larvae per strain). The average CFU for the injected wild‐type cells in the three experiments was 8.2 × 10^3^ and 9.5 × 10^3^ for *∆arpA* mutant. Infected larvae were incubated at 37°C for up to 5 days in a humidified incubator and mortality was assessed at daily intervals.

### 2.8 Construction of an *arpR* expression plasmid

The full‐length *arpR* gene including 146 bp of sequence upstream from the GTG start codon and 71 bp downstream from the stop codon were amplified from AB5075 genomic DNA by PCR (Phusion Hot‐Start Polymerase, Thermo Scientific). Primers arpR Exp.1 (5′‐CATTTAAATCGCTTATAACAC‐3′) and arpR Exp.2 (5′‐TTATCGCTCTTATTCAAACT‐3′) were phosphorylated prior to PCR amplification to add 5′ phosphates with T4 polynucleotide kinase (New England Biolabs). The *arpR* fragment was gel purified and ligated (Fast‐Link Ligation Kit, Epicentre Biotechnologies) into pWH1266 that had been digested with ScaI and treated with recombinant shrimp alkaline phosphatase (rSAP, New England Biolabs). The ligation was electroporated into competent *E. coli* EC100D Transformax cells (Epicentre Biotechnologies). Plasmids that conferred tetracycline resistance but not ampicillin resistance were purified and confirmed by DNA sequencing prior to introduction into *A. baumannii*. This produced the expression vector parpR.

### 2.9 Switching frequency determination

Cultures were grown to the OD_600_ ~0.7 or ~1.7 in LB medium, serially diluted in LB, and plated on 0.5× LB, 0.8% agar. Plates were incubated at 37°C overnight. CFU per milliliter and number of variant colonies were determined for each strain and variant.

### 2.10 RNA isolation

Cultures of *A. baumannii* strain AB5075 and the *ΔarpR* mutant were grown in LB medium at 37°C with shaking to an OD_600_ ~0.7. Cultures of *A. baumannii* strain AB5075 harboring pWH1266 or parpR/WH1266 were grown in LB medium supplemented with tetracycline (5 μg/ml) at 37°C with shaking to an OD_600_ ~0.5. Cells were harvested from cultures by centrifugation and RNA was isolated with the MasterPure RNA Purification Kit according to the manufacturer's protocol (Epicentre). Contaminating DNA was removed by treatment with TURBO DNA‐free according to the manufacturer's protocol (Ambion). DNA contamination was evaluated by PCR with purified RNA as template and RNA concentration was quantified with a NanoDrop ND‐1000 spectrophotometer.

### 2.11 Quantitative real‐time PCR

Total RNA (1 μg) purified from strains AB5075 with or without plasmid and *ΔarpR* were converted into cDNA by using the iScript cDNA synthesis kit (Bio‐Rad) with random primers and SuperScript III reverse transcriptase (Invitrogen). Cycling parameters for cDNA synthesis were as follows: 25°C for 5 min, 42°C for 45 min, and 85°C for 5 min. cDNA reactions were then diluted 1:10 with sterile H_2_O supplemented with 10 μg/ml yeast t‐RNA (Roche). Diluted cDNA was used as template for experimental reactions. Oligonucleotide primer pairs for quantitative real‐time PCR (qRT‐PCR) were generated by the Primer‐BLAST program available at www.ncbi.nlm.nih.gov/tools/primer-blast/. Primers were designed to amplify a 145‐bp fragment of clpX (clpX qRT For 5′‐GCGTTTGAAAGTCGGGCAAT‐3′, clpX qRT Rev 5′‐CCATTGCAAACGGCACATCT‐3′) and a 151‐bp fragment of arpA (arpA qRT 1 5′‐TCGCGTACATATCCGGCAAA‐3′, arpA qRT 2 5′‐GGCAAGCGGCTTATCAACTG‐3′). qRT‐PCR was performed using iQ SYBR Green Supermix (Bio‐Rad) with Bio‐Rad CFX Connect cycler. The following cycle parameters were utilized to amplify and quantify fragments: 95°C for 3 min and then 95°C for 10 s, 55°C for 10 s, and 72°C for 20 s, repeated 40 times. Melt curve data were collected to ensure proper amplification of target genes. Data were generated from three separate RNA isolation and cDNA preparations and at least two technical replicates for each primer set. Relative expression of each gene was determined by comparing target gene expression with control gene (*clpX*) expression using the Pfaffl method (Pfaffl, 2001).

### 2.12 Antimicrobial resistance assays

Strains to be tested were grown to an OD_600_ of 0.3 and then diluted to an OD_600_ of 0.05 with sterile LB. The minimum inhibitory concentration for various antibiotics was determined using E‐test strips on LB agar plates according to the manufacturer's instructions (Biomerieux). The MICs for each antibiotic were determined after 12 hr of growth at 37°C. All susceptibility tests were done in duplicate.

## 3 Results

### 3.1 An RND‐type efflux system is required for the opaque to translucent colony opacity phase variation

**Figure 1:**
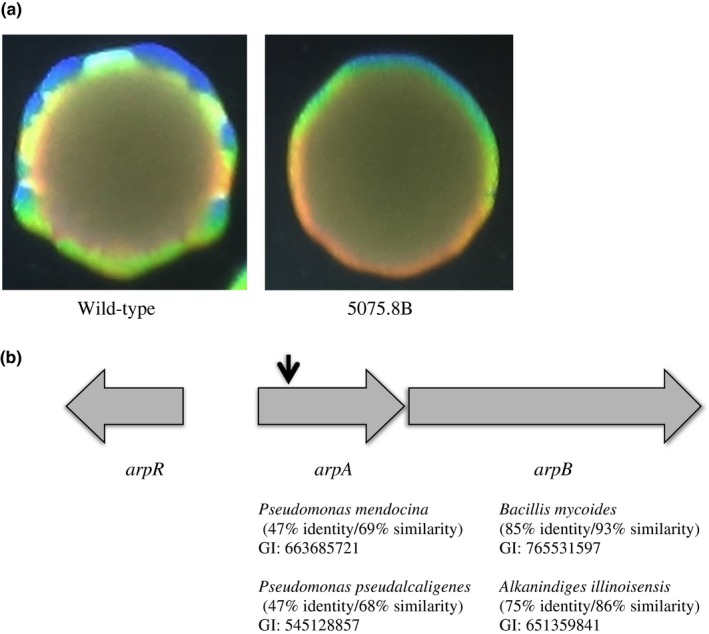
Decreased phase variation in an *arpA* mutant. (a) A typical wild‐type opaque colony variant of AB5075 is shown compared to 5075.8B *arpA::* EZ‐Tn5 <Tet‐1> after 36 hr of growth on a 0.5× LB, 0.8% agar plate. The mottled appearance at the outside edge of the wild type is due to translucent variants arising within the opaque colony. (b) The organization of the *arpAB* region and the site of the *arpA::* EZ‐Tn5 <Tet‐1> insertion that blocks phase variation is depicted by an arrow. Proteins exhibiting the closest match to ArpA and ArpB are shown below each gene

Previously, our laboratory reported on a phase variable mechanism in *A. baumannii* that results in the interconversion between opaque and translucent colonies (Tipton et al., 2015). This phase variation was stimulated at high density and when opaque colonies are grown for 36–48 hr, they become highly mottled in appearance due to translucent variants arising at high frequency within the colony (Figure 1a). This mottled appearance formed the basis for a genetic screen to identify mutants with a reduced frequency of phase variation in the opaque to translucent direction, as these mutants would have a reduction or absence in the mottled appearance of the colony. A library of EZ‐Tn5 <Tet‐1> insertions in AB5075 was screened for colonies that did not exhibit the mottled appearance after 36 hr of growth and mutants with this phenotype were isolated and confirmed by subsequent replating. One mutant 5075.8B (Figure 1a) was characterized further and the transposon insertion was mapped to nucleotide 64 of a 1,101 nucleotide open reading frame (ABUW_0034) encoding a putative periplasmic membrane fusion component of RND‐type efflux systems (Figure 1b). This protein exhibited the highest degree of identity to annotated RND‐type transporters from *Pseudomonas mendocina* (47% identity/69% similarity) and *Pseudomonas pseudoalcaligenes* (47% identity/68% similarity). A second open reading frame (ABUW_0035) was present beginning 3 bp downstream of ABUW_0034 that encoded a putative inner membrane transporter component of RND‐type systems. This protein exhibited the highest identity to an annotated RND‐type transporter from *Bacillus mycoides* (85% identity/93% similarity) and to *Alkanindiges illinoisensis* (75% identity/86% similarity). Given their close proximity, these two genes likely formed an operon and were previously identified in *A. baumannii* as a locus upregulated in the presence of farnesol and were designated as *acrAB* (Kostoulias et al., 2016). However, the overall similarity of these proteins to the *E. coli* AcrA (23% identity/39 similarity) and AcrB (25% identity/43% similarity) was low and there are other RND‐type systems in *E. coli* that exhibit greater identity to the *A. baumannii* proteins. In addition, as outlined below, this RND system confers aminoglycoside resistance, therefore, these genes were designated *arpA* and *arpB* (aminoglycoside resistance pump). The frequency of phase variation from opaque to translucent was quantitated in individual colonies of the 5075.8B mutant and wild‐type cells after 48 hr of growth, which revealed an average 55‐fold decrease in the opaque to translucent switch in the *arpA*:: EZ‐Tn5 <Tet‐1> mutant 5075.8B. While this analysis was being conducted, it was observed that there was significant colony to colony variation within the same strain with respect to phase variation frequencies, leading to large standard deviations. As a result of this, broth grown cells were used for subsequent experiments, which did reduce variability, although significant variability still existed. In broth cultures, the phase variation rate of the 5075.B mutant was 77‐fold lower than wild type (Table 1).

**Table 1:** Phase variation frequencies

Strain	Relative O to T phase variation frequency^a^
AB5075 wild type	1^b^
5075.8B *arpA1*::EZTn‐5Tc	0.013 ± 0.02 (77‐fold decrease)
KT2 ∆*arpA2*	0.014 ± 0.03 (71‐fold decrease)
AB00075 *arpB::Tc*	0.0013 ± 0.001 (769‐fold decrease)
KT3 ∆*arpR*	0.0009 ± 0.0002 (1,916‐fold decrease)

Strain	Relative T to O phase variation frequency^a^
AB5075 wild type	1^c^
KT2 ∆*arpA2*	1.5 ± 0.6
AB00075 *arpB::Tc*	1.2 ± 0.5
KT3 ∆*arpR*	N.D.

a Determined in cultures at an OD_600_ of 1.85–1.9.

b Phase variation frequency from O to T was typically 25%–35%.

c Phase variation frequency from T to O was typically 40%–50%.

To verify the role of the *arpA* gene in phase variation and to avoid polar effects on the downstream *arpB* gene that likely occurred from the *arpA*:: EZ‐Tn5 <Tet‐1> insertion, an in‐frame deletion in *arpA* (∆*arpA2*) was constructed. This mutation had a similar effect on phase variation as the original *arpA1*::EZTN<Tc> mutant, where the frequency of switching from opaque to translucent was reduced 71‐fold in cultures at an OD_600_ = 1.9 (Table 1). This ∆*arpA2* mutant strain was designated KT2.

Complementation analysis was performed to determine if the loss of *arpA* was responsible for the phase variation defect. When a plasmid containing the *arpA* gene was introduced into the *arpA* deletion mutant (KT2), colonies regained the ability to form the mottled phenotype due to translucent variants arising at high frequency (data not shown). In addition, the frequency of phase variation in 24 hr colonies was increased by 3,600‐fold in the *arpA* deletion mutant containing the cloned *arpA* gene (parpA), relative to the *arpA* deletion mutant containing the pWH1266 vector alone (Figure S1, panel A).

### 3.2 An *arpB* mutation also decreases the opaque to translucent phase variation

The gene located immediately downstream from *arpA* is predicted to encode a putative inner membrane transporter of RND efflux systems and likely functions with ArpA in this process. This gene was designated *arpB*. To determine if *arpB* also regulates phase variation, two separate transposon insertions in *arpB* (AB00075, AB00076) were obtained from the *A. baumannii* AB5075 transposon insertion library at the University of Washington (Gallagher et al., 2015). Like the *arpA* mutant, opaque colonies of the *arpB* mutants did not produce mottled colonies at 48 hr. The frequency of phase variation for AB00075 *arpB::Tc* in broth grown cells at an OD_600_ of 1.9 was reduced an average of 769‐fold compared to wild‐type cells (Table 1).

### 3.3 *arpA* and *arpB* mutations do not alter the translucent to opaque switch

The effect of the ∆*arpA* mutation on the reciprocal opacity switch, from translucent to opaque, was measured in KT2 grown to an OD of 1.9, where the rate of phase variation was increased 1.5‐fold over wild type (Table 1). The *arpB::Tc* mutant AB00075 exhibited a phase variation rate for translucent to opaque that was 1.2‐fold greater than wild type.

### 3.4 An *arpB* mutant still produces an extracellular signal that stimulates phase variation

Previous work demonstrated that the increase in phase variation at high cell density is mediated, in part, by the accumulation of a secreted signal (Tipton et al., 2015). If this signal is dependent on ArpAB for secretion and sensed at the cell surface or the cytoplasmic membrane, then this could explain the reduction in phase variation in the *arpA* mutant. To test this possibility, conditioned media were prepared from opaque variants of wild‐type and the ∆*arpB* mutant at an optical density of 1.7 and tested for the ability to stimulate phase variation in the opaque to translucent direction. Conditioned media from wild‐type cells stimulated the opaque to translucent conversion 11‐fold and the conditioned media from the *arpB* mutant stimulated phase variation ninefold (Table 2). There was not a statistically significant difference between these values (*p *=* *.39) indicating that the *arpB* mutant produced a similar level of extracellular signal activity as the wild‐type parent.

**Table 2:** Effect of conditioned media on relative phase variation frequency

Growth condition^a^	Opaque to translucent switching frequency
LB broth	0.35% ± 0.29
Wild‐type opaque conditioned media	3.9% ± 2.8 (11‐fold increase)
∆*arpB* opaque conditioned media	3.2% ± 2.7 (9‐fold increase)

a Determined in cells grown to an optical density A_600_ of 1.0.

### 3.5 Role of ArpAB in antimicrobial resistance

It is well established that RND‐type systems can export antimicrobials and confer higher levels of resistance (Alvarez‐Ortega et al., 2013; Anes et al., 2015; Coyne et al., 2011; Magnet et al., 2001; Rosenfeld et al., 2012; Routh et al., 2011; Venter et al., 2015). Therefore, both the *arpA* and *arpB* mutants were tested for levels of resistance to various antibiotics. For this analysis, both the opaque and translucent variants of each mutant and wild‐type cells were tested (Table 3). Due to the multiple resistances that already exist in AB5075, the number of antibiotics that could be tested was limited. There were no significant differences between wild‐type and the *arpA* mutant for any of the antibiotics tested (Table 3), although there were subtle differences between opaque and translucent variants of wild type as reported previously (Tipton et al., 2015). However, the *arpB* mutant AB00075 was more sensitive to the two aminoglycosides that were tested, amikacin and tobramycin (Table 3). This sensitivity was seen in both the opaque and translucent variants of AB00075 *arpB::Tc*.

**Table 3:** Minimum inhibitory concentrations for antibiotics

Strain	MIC (μg/ml)					
	COL	TET	AK	RIF	TIG	TOB
WT opaque	0.38	3	128	3	3	96
∆*arpA* opaque	0.38	3	128	3	3	96
*arpB::Tc* opaque	0.38	ND	64	3	3	24
WT translucent	0.38	3	64	4	3	24
∆*arpA* translucent	0.38	3	64	4	3	24
*arpB::Tc* translucent	0.38	ND	16	4	3	8

COL, colistin; TET, tetracycline; AK, amikacin; RIF, rifampicin; TIG, tigecycline; TOB, tobramycin.

### 3.6 *arpA* and *arpB* mutations selectively increase surface motility in the opaque variants

**Figure 2:**
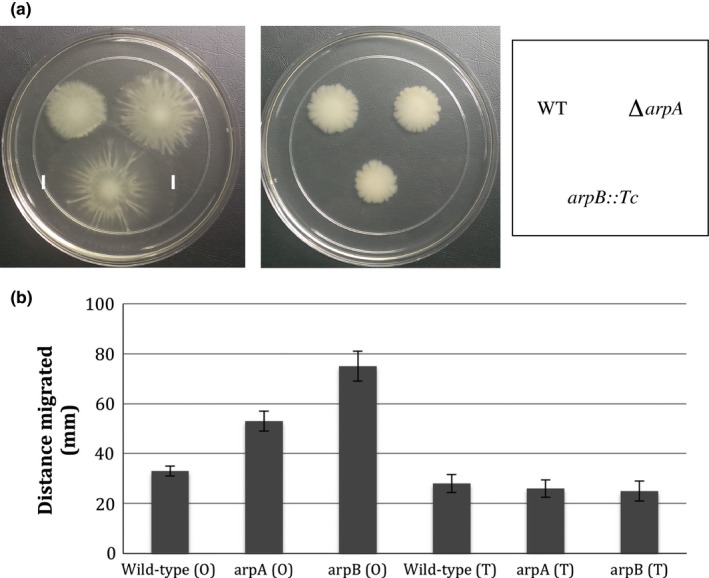
Surface motility. (a) The motility of opaque (left panel) and translucent (right panel) variants of wild‐type AB5075, the ∆*arpA* mutant KT2, and *arpB::Tc* mutant AB00075 are shown after 12 hr of growth on 0.35% Eiken agar plates incubated at 37°C. (b) Quantitation of surface motility. The values shown represent the average of three separate motility assays for each strain. Conditions for the motility assays were the same as shown in A

Both the *arpA* and *arpB* mutants formed irregular, slightly spreading colonies on 0.8% agar plates that were distinct from wild‐type colonies that contained smooth rounded edges. This observation suggested that motility may be enhanced by the *arpA* and *arpB* mutations. To test this hypothesis, the motility of both opaque and translucent *arpA* and *arpB* mutants were compared to opaque and translucent wild‐type AB5075 on 0.3% Eiken agar plates. In opaque colonies, both the *arpA* and *arpB* mutants exhibited increased motility, 53 mm and 75 mm, respectively, over wild‐type value of 33 mm (Figure 2). However, in the translucent form, there was no significant difference in motility between wild‐type and either the *arpA* or *arpB* mutants (Figure 2). The increased motility observed in the *arpA* mutant was restored back to wild‐type levels by parpA containing the cloned *arpA* gene relative to cells containing the pWH1266 vector alone (Figure S1, panel B).

### 3.7 ArpAB is required for virulence in *Galleria mellonella*

**Figure 3:**
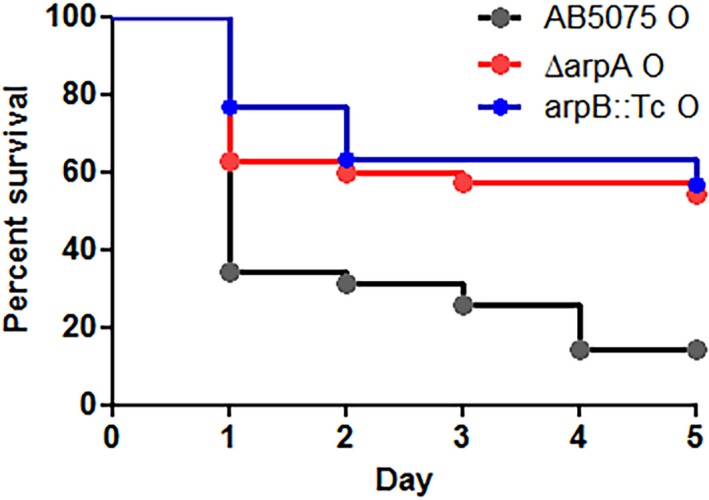
*arpA* and *arpB* mutants exhibit decreased virulence in a *Galleria mellonella* model. The ability of wild‐type AB5075 and the isogenic ∆*arpA* and *arpB::Tc* mutants to kill *G. mellonella* waxworms is shown on the Kaplan–Meier plots. The data shown represent the average of three independent experiments with a total of 30 worms per strain

RND‐type efflux systems have been shown to have roles in virulence in a number of bacterial pathogens (Alvarez‐Ortega et al., 2013; Delmar et al., 2014; Routh et al., 2011; Taylor, Bina, & Bina, 2012; Venter et al., 2015; Warner et al., 2007; Yoon et al., 2015). Therefore, the impact of *arpA* and *arpB* mutations on virulence were examined in a *Galleria mellonella* waxworm model that has previously been shown to be a useful model of virulence in *A. baumannii* (Esterly et al., 2014; Gaddy et al., 2012; Heindorf, Kadari, Heider, Skiebe, & Wilharm, 2014; Iwashkiw et al., 2012; Jacobs et al., 2014; Nwugo et al., 2012; Peleg et al., 2009; Repizo et al., 2015; Stahl, Bergmann, Gottig, Ebersberger, & Averhoff, 2015; Tipton et al., 2015). Since previous work indicated that the opaque form was more virulent, these studies were done with the opaque forms of wild‐type AB5075, KT2 ∆*arpA* and the *arpB::Tc* mutant (Tipton et al., 2015). Relative to wild‐type AB5075, both the ∆*arpA* and *arpB* mutations substantially reduced the ability of *A. baumannii* to kill *G. mellonella* waxworms (Figure 3a). The decreased virulence exhibited by the *arpA* mutant was restored by parpA containing the cloned *arpA* gene relative to cells containing the pWH1266 vector alone (Figure S1, panel C).

### 3.8 A divergently transcribed gene encoding a TetR‐type regulator represses *arpAB* and is required for the opaque to translucent switch

**Figure 4:**
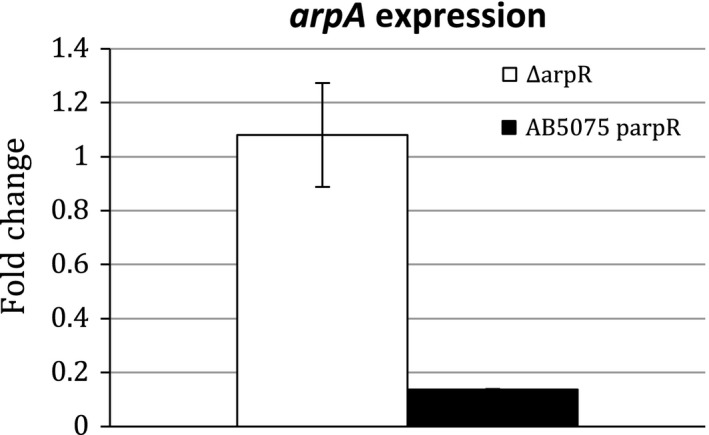
Effect of ArpR on *arpA* expression. The levels of *arpA* expression were determined by quantitative RT‐PCR and the values shown are relative to the control gene *clpX*. The values for the left side represent the levels of *arpA* expression in the *arpR* mutant relative to wild type. The values on the right side represent the levels of *arpA* expression in cells overexpressing *arpR* from a plasmid relative to cells containing the vector alone

Adjacent to the *arpAB* genes in *A. baumannii* was a divergently transcribed gene encoding a predicted TetR‐type repressor of the AcrR family (Figure 1). In *E. coli*, the AcrR protein acts to repress the *acrAB* operon, which is organized in a similar manner as *arpAB*. To determine if a similar function was present in *A. baumannii*, an in‐frame deletion was constructed in this gene, designated *arpR*. The ∆*arpR* deletion did not have a significant impact on *arpAB* expression, with the levels of expression 1.1‐fold higher in the mutant (Figure 4). However, the *arpR* mutation reduced the frequency of the opaque to translucent conversion by 1,916‐fold (Table 1).

As a second method to determine if ArpR could function as a repressor, the *arpR* gene was overexpressed by cloning the gene into pWH1266, where expression was driven from the promoter for the β‐lactamase gene. When this plasmid was introduced into wild‐type AB5075, the colony morphology was altered with irregular slightly spreading colonies that were similar to *arpA* and *arpB* mutants, suggesting that expression of both *arpA* and *arpB* were reduced when ArpR was overexpressed. To investigate this possibility, expression of the first gene *arpA* was examined by qRT‐PCR and was found to be 7.3‐fold lower in cells with parpR versus the pWH1266 vector alone (Figure 4). Next, we investigated if the reduced ArpAB expression resulting from ArpR overexpression impacted the opaque to translucent switch. In cells overexpressing the *arpR* gene, the frequency of phase variation was reduced threefold, relative to cells containing the vector only (data not shown).

## 4 Discussion

In this study, a previously undescribed RND‐type efflux system in *A. baumannii* was found to regulate phase variation in the opaque to translucent direction. The original mutation defining this phenotype was a transposon insertion in *arpA*, a gene encoding a membrane fusion component of a putative RND‐type efflux system. This *arpA* mutation resulted in a 77‐fold decrease in the frequency of phase variation from the opaque to translucent colony opacity phenotype (Table 1). The role of *arpA* in this process was confirmed by the construction of a nonpolar, in‐frame deletion within *arpA*, which decreased the conversion to translucent colonies by 71‐fold, and by complementation analysis with the wild‐type *arpA* gene. In addition, a transposon insertion in the downstream *arpB* gene obtained from the *A. baumannii* AB5075 transposon library at the University of Washington (Gallagher et al., 2015) exhibited a stronger effect with a 769‐fold reduction in phase variation (Table 1). Interestingly, neither the *arpA* or *arpB* mutations had a significant effect on phase variation in the translucent to opaque direction. This provides strong evidence that a distinct mechanism regulates phase variation in each direction.

Two additional phenotypes were altered by the loss of *arpA and arpB*. First, surface motility of the opaque variants on 0.3% agar plates was increased, with the *arpB* mutant exhibiting a more substantial increase than the *arpA* mutant (Figure 2). Interestingly, in translucent variants, the motility of both the *arpA* and *arpB* mutants was similar to wild‐type. The basis for the selective motility increase in opaque variants is unknown, but previously it was noted that in wild‐type cells, the opaque variants were more motile (Tipton et al., 2015). Although the actual mechanism responsible for motility in AB5075 has not been identified, if this mechanism was only expressed in opaque cells and also repressed by ArpAB, it could explain the selective increase in motility seen in *arpA* or *arpB* mutants in the opaque background. Second, the *arpA* mutation decreased the ability of *A. baumannii* AB5075 to kill *G. mellonella* waxworms. Additional examples exist where the loss of an RND‐type system decreases virulence (Alvarez‐Ortega et al., 2013; Delmar et al., 2014; Routh et al., 2011; Taylor et al., 2012; Venter et al., 2015; Warner et al., 2007; Yoon et al., 2015). The decreased virulence may be due to a role for ArpAB in efflux of host antimicrobial peptides or other antibacterial compounds present inside *G. mellonella*.

The *arpB* mutant consistently exhibited stronger effects than the *arpA* mutant for phase variation (Table 1), surface motility (Figure 2), and antibiotic sensitivity (Table 3). It is unlikely that this results from the *arpA* mutation being leaky, as the in‐frame deletion removed most of the coding region. The weaker phenotype of the *arpA* mutation may indicate that a periplasmic fusion protein from another RND‐type system can partially substitute for ArpA. The stronger effect of a *arpB* mutation suggests that ArpB is unable to be substituted for by another RND component and has a more critical role than ArpA. However, this hypothesis is speculative at the current time. In addition, the outer membrane channel that works with ArpAB is unknown, but AbuO, a TolC‐like protein is a possible candidate (Srinivasan, Vaidyanathan, & Rajamohan, 2015).

A TetR‐type transcriptional regulator was encoded adjacent to the *arpAB* genes and divergently transcribed, a genetic organization similar to that of *acrR‐acrAB* in *E. coli* and *mexR‐mexAB* in *P. aeruginosa*. A ∆*arpR* mutation did not alter *arpAB* expression, however, overexpressing the *arpR* gene decreased *arpAB* expression by 7.3‐fold. In addition, cells overexpressing *arpR* exhibited phenotypes that at least partially mimicked the *arpAB* mutations, as the colonies formed the irregular spreading appearance and the frequency of the opaque to translucent conversion was reduced threefold. It is unclear why the effects of ArpR on *arpAB* expression are only seen when it is overexpressed, but under the laboratory growth conditions we tested, it does not have a role in *arpAB* regulation, possibly because it is not expressed at high enough levels to mediate repression. If certain environmental conditions increased *arpR* expression, this condition would be predicted to repress *arpAB* and reduce the opaque to translucent conversion.

To our knowledge, this represents the first report of an RND‐type efflux system regulating bacterial phase variation. A direct role for ArpAB in this process is unlikely and loss of ArpAB may indirectly modulate a regulatory pathway controlling phase variation. At the present time, the regulatory mechanism regulating the interconversion between opaque and translucent colonies is unknown. However, whole genome sequencing suggests that nucleotide changes are not responsible. This information, together with the observation that phase variation between colony opacity phenotypes is essentially undetectable in cells at low density, but is sharply activated at high density and reaches frequencies above 10% (Table 1) suggests a nonmutational mechanism is involved.

A possible mechanism to control phase variation is via a bistable switch involving one or more regulatory proteins (Casadesus & Low, 2013; Chang et al., 2010; Dubnau & Losick, 2006; Ferrell, 2002; Maamar & Dubnau, 2005; Mitrophanov & Groisman, 2008; Piggot, 2010; Turner, Vallet‐Gely, & Dove, 2009; Veening, Smits, & Kuipers, 2008). Bistability has previously been shown to regulate colony opacity in *Pseudomonas fluorescens* (Gallie et al., 2015). Based on this information, there are several possibilities for how ArpAB might regulate phase variation. First, the extracellular signal that stimulates the opaque to translucent phase variation might require ArpAB for secretion. This would imply that the signal works via a sensing mechanism that operates at the cell surface or at the cytoplasmic membrane and then regulates the bistable switch controlling the phase variation. However, spent culture supernatants from an *arpB* mutant and wild‐type cells appear to activate the opaque to translucent switch at equal frequencies (Table 2). A second possibility is that a metabolite normally secreted by the ArpAB system accumulates in *arpAB* mutants and this alters a regulatory pathway that controls phase variation. The isolation of both extragenic and high‐copy suppressors that restore phase variation to an *arpA* or *arpB* mutant should help uncover the role of this efflux system in regulating the process of colony opacity phase variation in *A. baumannii* and these studies are in progress.

## Conflict of Interest

None declared.
