# Supplementary material for: Multiple roles for a novel RND‐type efflux system in Acinetobacter baumannii AB5075
Source: Microbiologyopen. 2016 Oct 19;6(2):e00418. doi: 10.1002/mbo3.418 (PMC5387308; doi:10.1002/mbo3.418)
Supplement: Supplementary file 1 [file MBO3-6-na-s001.pdf]

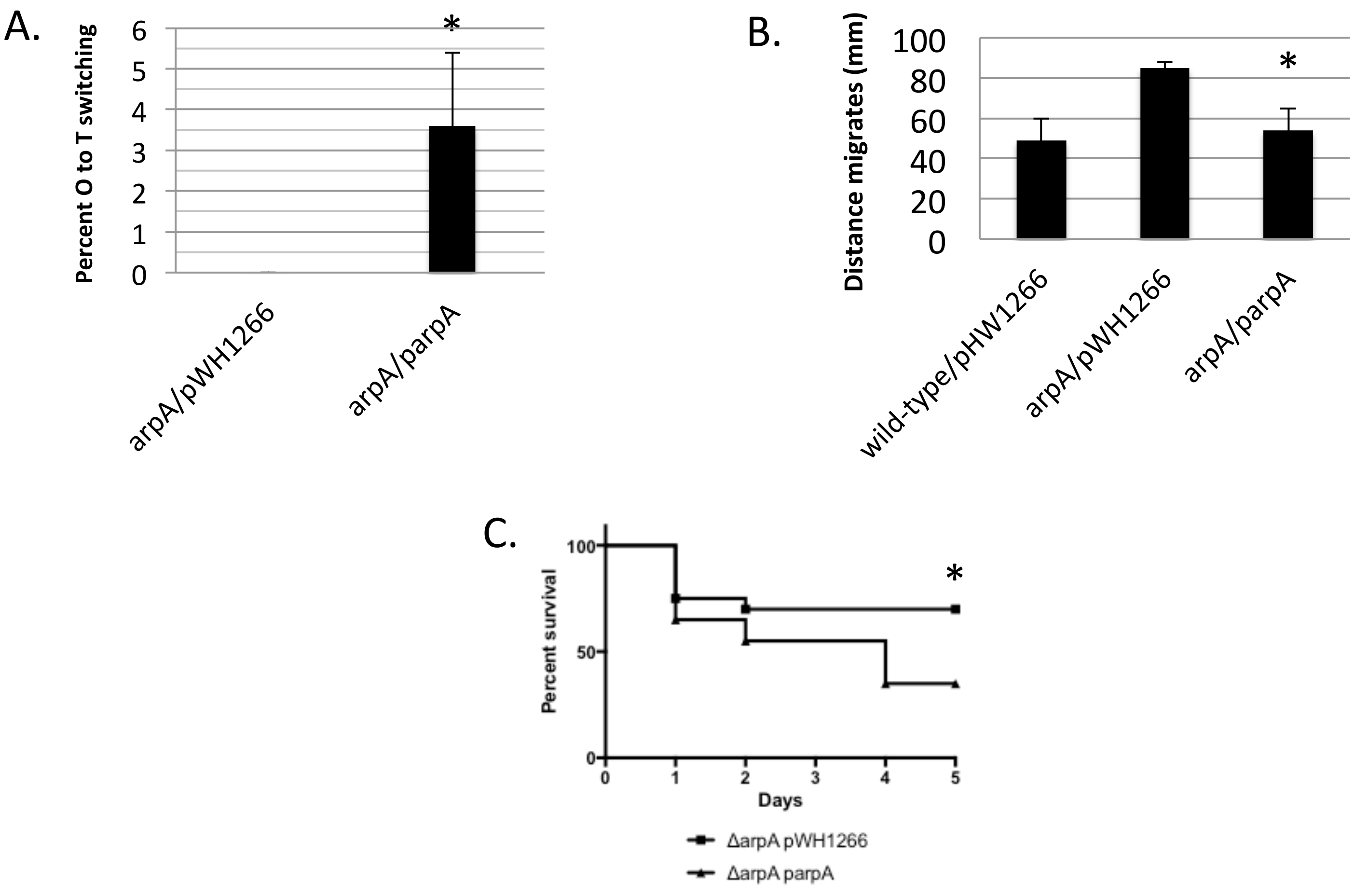

Supplemental Figure 1. In panel A, the opaque to translucent switching frequency was determined in 24 hr. old colonies of the *ΔarpA* mutant containing the pWH1266 vector alone or with pWH1266 containing the cloned *arpA* gene (parpA). Panel B, motility of the indicated strains on 0.3% Eiken agar plates after 14 hours of growth is shown. Panel C, the ability of the indicated strains to kill *Galleria mellonella* waxworms was determined at daily intervals. For each strain, 20 waxworms were used. For all the experiments shown in panels A-B, the difference between *ΔarpA* mutant cells containing the pWH1266 vector alone and parpA was statistically significant (p = <0.05) and indicated with an asterisk.
